# Linking Industrial Hazards and Social Inequalities: Environmental Injustice in Gujarat, India

**DOI:** 10.3390/ijerph16010042

**Published:** 2018-12-25

**Authors:** Jayajit Chakraborty, Pratyusha Basu

**Affiliations:** Department of Sociology & Anthropology, University of Texas at El Paso, El Paso, TX 79968, USA; pbasu@utep.edu

**Keywords:** environmental justice, industrial pollution, toxic chemicals, economic development, emerging economy, India

## Abstract

Industrial development in India has rarely been studied through the perspective of environmental justice (EJ) such that the association between industrial development and significant economic and social inequalities remains to be examined. Our article addresses this gap by focusing on Gujarat in western India, a leading industrial state that exemplifies the designation of India as an “emerging economy.” We link the geographic concentration of industrial facilities classified as major accident hazard (MAH) units, further subdivided by size (large or medium/small) and ownership (public or private), to the socio-demographic composition of the population at the subdistrict (taluka) level. Generalized estimating equations (GEEs) are used to analyze statistical associations between MAH unit density and explanatory variables related to the economic and social status of the residential population at the subdistrict level. Our results indicate a significant relationship between presence of socially disadvantaged populations (Scheduled Castes and Scheduled Tribes) and density of all types of MAH units, except those associated with the public sector. Higher urbanization and lower home ownership are also found to be strong predictors of MAH unit density. Overall, our article represents an important step towards understanding the complexities of environmental inequalities stemming from Gujarat’s industrial economy.

## 1. Introduction

Environmental injustices in India have most often been discussed in terms of extreme events (e.g., the 1984 Bhopal Gas Tragedy) or struggles launched by social movements (e.g., against bauxite mining in Niyamgiri hills, Orissa) [1]. In the process, chronic contexts of pollution that are not characterized by visible forms of resistance often receive less attention even as pollution maybe equally, or even more, inequitably distributed here. This argument especially holds true for hazardous industrial pollution which needs to be studied in terms of the social characteristics of the population that resides in industrial regions and is likely to be exposed to the harmful effects of hazardous waste production.

India’s position as an “emerging economy” is partly linked to its industrial performance [2,3], and industrialization here has a highly uneven distribution across the country [4]. Gujarat in western India is one of the leading states in terms of industrial production [5], as well as one of the top states in terms of the production of hazardous waste [6]. A number of studies have focused on explicating the causes of successful industrial development in Gujarat [7,8,9] or drawn attention to deepening economic and social polarization within the state [10,11]. A distributive environmental justice (EJ) analysis of hazardous industrial pollution would contribute to this body of research by linking the spatial patterns of industrial development with social inequalities.

This article aims to address an important research gap in EJ studies in India, as well as contribute more broadly to studies of industrial development, by analyzing the relationship between the spatial distribution of hazardous industrial facilities classified as major accident hazard (MAH) units and pertinent socio-demographic factors in the highly industrialized state of Gujarat. Specifically, the objective is to determine if socially disadvantaged communities in Gujarat reside disproportionately in areas burdened by higher densities of MAH units, further subdivided by the type of MAH unit as defined by capacity (large versus medium/small) and sector (private versus public). Data utilized for this study combines the locations and characteristics of Gujarat’s MAH units in 2014 with population and housing information obtained from the 2011 Census of India. The unit of analysis is the subdistrict (known as *taluka* in Gujarat), an administrative division in India below the state and district levels, and the smallest geographic unit for which data on population and housing characteristics pertinent to our study are available in the 2011 Census. The subdistrict becomes a useful unit of analysis for Gujarat due to the concentration of industrial development around urban centers which could become masked at the relatively large scale of the district. Our statistical analysis is based on generalized estimating equations (GEEs) that account for geographic clustering of subdistricts within districts and provide statistically valid insights on the association between MAH unit density and specific socio-demographic characteristics of the population. Overall, our study becomes significant for gaining a better understanding of the negative aspects of industrialization in Gujarat to balance against the often unequivocal highlighting of positive aspects in governmental and corporate reports [5,12].

## 2. Materials and Methods

### 2.1. Study Area

With a total population of 60,439,692, Gujarat ranks as the tenth most populous among India’s 28 states, and it is the seventh largest state in terms of area encompassing 196,024 km^2^ [13]. Around 42.6% of Gujarat’s population resides in urban areas, which is higher than the 31.2% urban population for India as a whole. Gujarat’s position as a leading industrial state in India is demonstrated by the fact that it contributed 18.4% of India’s total industrial output in 2017, the largest share among all states in India [5]. It has also become a favored destination for Foreign Direct Investment (FDI) [5,12] through the holding of biennial “Vibrant Gujarat Summits” that showcase the business-friendly policies of the state [14,15]. However, as mentioned above, this narrative of industrial success needs to be qualified. First, Gujarat is also one of the leading states in terms of hazardous industrial waste production, generating about 28.8% of India’s total industrial hazardous waste production (40.6% of total land disposable hazardous waste) [16,17]. It also contains about 30.0% of all MAH units in the country [18]. Second, social inequalities remain a key concern in the state both due to religious violence, as well as widening income inequalities [10,11,19].

Industrialization has a long history in Gujarat and is famously associated with cotton textile production in Ahmedabad, the largest city in terms of population and Gujarat’s main financial center [20]. More recently, textiles have declined in importance and chemical industries have become a leading sector [7], with the development of offshore oil and gas production contributing to the rising importance of petrochemicals. This shift has also changed the geography of industrial growth in the state. While the districts of Ahmedabad and Vadodara in central Gujarat and Surat and Bharuch in south Gujarat have been centers of industrial growth since the 1960s, this has now extended to include a coastal belt associated with petrochemical and port development, especially in south and west Gujarat [6,14]. Industrial growth has also been spurred in new areas through the establishment of special economic zones (SEZs), and the growth of SEZ-related industries is especially prominent in the previously underdeveloped northwestern district of Kutch [21,22]. The problem of hazardous waste production thus reflects both historical patterns and new geographies of industrialization, as Bharuch and Ahmedabad districts are the highest and second highest generators respectively of hazardous industrial waste in India, and Vadodara and Kutch districts are nationally ranked among the top ten districts producing hazardous industrial waste [16]. These trends highlight the growing need to examine the distributive EJ implications of industrialization in Gujarat.

### 2.2. Dependent Variables

A major accident hazard (MAH) unit is an industrial facility in which an operation or process is carried out that involves or is likely to involve (including through on-site storage or transport, isolated storage, or transport via carrier or pipeline) one or more hazardous chemicals in quantities equal to or in excess of threshold quantities [23]. The regulated hazardous chemicals and their threshold quantities are enumerated in the Manufacture, Storage and Import of Hazardous Chemical Rules 1989, which functions under the broader rubric of the Environment (Protection) Act 1986 promulgated in the aftermath of the catastrophic Bhopal Gas Tragedy of 1984 [23]. Data on MAH units in Gujarat is available through the Director Industrial Safety and Health, Labour and Employment Department, Government of Gujarat [24]. Our dataset included geographic coordinates of 402 MAH units in Gujarat that were functioning in 2014, as well as their names (owner of company), sector (public or private), and production capacity (large, medium, or small) and was purchased from ML InfoMap [25] through Lead Dog Consulting [26]. Several EJ studies in the U.S. have relied on more sophisticated measures of environmental health risk that are based on the type, volume, or toxicity of industrial toxic releases [27]. However, facility-specific data on hazardous chemical emissions, toxicity, waste generation, or other indicators of human health risk are not currently available for industries in Gujarat or elsewhere in India. Consequently, our analysis is limited to MAH units, which comprise industrial establishments dealing with the most hazardous chemicals, and specific subsets of these units. In terms of production capacity or size, these hazardous industries were classified as either large (*n* = 307) or medium to small (*n* = 87). In terms of sector of ownership, these industries were classified as either private (*n* = 307) or public (*n* = 79).

This study models risks from exposure to hazardous industries by calculating the density of industrial facilities classified as MAH units in each subdistrict in Gujarat. Our approach to estimating hazardous industry density is conceptually similar to that used in previous EJ research to calculate the density of point sources of environmental pollution [28,29,30,31], because it measures the clustering of MAH units and accounts for the fact that some of these facilities are located near the boundaries of subdistricts. While the density of MAH units is not a direct indicator of toxic exposure or human health risk, it does represent the relative concentration of such industries at the subdistrict level.

The density of all MAH units and specific types of MAH units was estimated for each subdistrict based on a technique for modeling point density described by Bailey and Gatrell [32] and used in EJ studies conducted in the U.S. [29,31]. This geographic information system (GIS)-based technique comprised the following steps:

1. A 2 km by 2 km spatial grid of points was overlaid on the map layer representing the geocoded locations of MAH units.
2. The total number of MAH units within a 5 km search radius of each grid point was calculated. The 5 km radius has been recommended as the maximum distance for adverse effects associated with point sources of environmental pollution [30,33].
3. The number of MAH units within 5 km of each grid point was divided by the area of the search to derive a MAH unit density value for every grid point.
4. The map layer representing subdistrict boundaries was overlaid on the grid of MAH unit density and the mean (average) density value of all grid points located within each subdistrict boundary was calculated and assigned as an attribute of that subdistrict.

These four steps were repeated using map layers for large capacity, medium/small capacity, private, and public industries, respectively. Descriptive statistics for the dependent or hazardous industry density variables for subdistricts in Gujarat are provided in Table 1.

### 2.3. Explanatory Variables

Inequalities in the distribution of MAH units in Gujarat were analyzed using five variables from the 2011 District Census Handbook—Part B [34] to capture the extent of economic development and social disadvantage, and one variable from the 2011 Houselisting and Housing Census Data [35] to assess home ownership rates. These variables have been utilized in previous EJ studies in India and thus are likely to be pertinent for Gujarat [36,37]. Descriptive statistics for the explanatory variables used in this study are summarized in Table 1.

Population density and the proportion of urban population in the subdistrict were used as control variables for our analysis, following previous EJ studies that used both these variables [37,38]. Population density can have both positive and negative effects on hazardous industry location patterns. Some EJ studies indicate that densely populated areas are likely to attract more pollution-generating activities [29,31,39], but other studies have shown that industries are often located in areas with empty space as government policies seek to deconcentrate hazardous industries in densely populated areas [40,41]. The proportion of urban population is estimated based on the population residing in census towns or statutory towns [42]. Census towns have a population of at least 5000 people, a density of population of at least 400 people per square kilometer, and at least 75% of main male workers engaged in non-agricultural occupations. Statutory towns are administered by a municipality, corporation, cantonment board, or notified area committee. MAH unit density can be hypothesized to be associated with urban concentration in one of two ways. A higher proportion of urban population can either serve to attract hazardous industries seeking access to labor and transportation [43], or repel them as urban dwellers organize to ensure that industrial pollutants are not produced in their immediate vicinity [44].

Social disadvantage is measured through variables denoting the proportions of Scheduled Caste (SC) and Scheduled Tribe (ST) populations, respectively. SC nomenclature refers to historically disadvantaged caste groups within Hindu, Buddhist, and Sikh religions who have faced social discrimination due to their lower caste status and associated occupational roles. ST groups often denote indigenous status in India, and display distinctive cultural histories (e.g., through their languages) and environmental practices (e.g., dependence on forest-based livelihoods). SCs and STs are listed in the Indian Constitution and provided special protections against discrimination, and in the case of STs, specific policies to maintain their cultures and environments. Both groups have access to reserved quotas in education and employment, which seek to enable their social and economic advancement. Existing studies have documented the economic and social marginalization of SC/ST groups [45,46] and the residential segregation of castes in urban India [47]. With regard to environmental inequality, previous national level research indicates that both SC and ST percentages are significantly higher in districts that generate industrial hazardous waste, compared to districts that do not produce such waste [37]. Our study seeks to analyze whether these distributive injustices exist for MAH units in the state of Gujarat. According to the 2011 Census, SCs comprised 6.7% and STs comprised 14.8% of Gujarat’s total population. Gujarat’s SC population is slightly more rural with 56.0% of SCs residing in rural areas [48], while the ST population is highly rural with 90.0% residing in rural areas [49]. SC and ST populations also differ in terms of geographic distribution within Gujarat: SC populations are found mostly in northern and western districts [48], while ST populations are located in hilly areas along the state’s eastern border and in its southern coastal districts [50].

Since income data is not published by the Census of India, we used two different variables to evaluate the socioeconomic status of subdistricts: literacy rate and home ownership rate. Literacy rate is counted for people aged seven years and above in India and can influence industrial density in two ways. It could make the local labor force more qualified for industrial employment (attract industries), as well as raise knowledge about and hence precipitate opposition to the adverse consequences of industrial pollution (repel industries). Home ownership rate, calculated as proportion of households in the subdistrict that owned a home, was also included as a socioeconomic indicator. This variable has been used frequently as an indicator of wealth and assets in other EJ studies, especially in the U.S. [31,39,51,52]. In a previous EJ study conducted in the city of Delhi [36], home ownership was found to have a non-significant relationship with exposure to outdoor air pollution, and our study of Gujarat contributes to ascertaining the significance of this variable in other contexts in India. It should be noted that the state of Gujarat (83.9%) ranks slightly below India as a whole (86.6%) in percentage of households owning a home, and slightly above India as whole in percentage of households renting their residence (13.5% for Gujarat compared to 11.1% for India) [53]. The lack of rental housing in India is viewed as impeding labor mobility and hence ease of access to industrial jobs [54].

### 2.4. Statistical Methodology

We first examined bivariate correlations between the density of each type of MAH unit (dependent variables) and each explanatory variable, at the subdistrict level. We then used a multivariate approach to analyze each of our five dependent variables as a function of all explanatory variables in a single model. Our multivariable models are based on generalized estimating equations (GEEs) with robust (i.e., Huber/White) covariance estimates, which extend the generalized linear model [55] to accommodate clustered data [56]. GEEs have been used extensively for analyses of clustered observations in the biological and epidemiological sciences [57,58], and more recently, in EJ studies conducted in the U.S. [52,59,60,61]. However, they have not been applied to analyze environmental inequalities in India where hazardous industries are concentrated primarily in urban areas and estimates of industrial facility density or waste production are likely to yield non-normal distributions [37].

GEEs are suitable for this study because they: (a) relax several assumptions of traditional regression models, (b) impose no strict distributional assumptions for the variables analyzed, and (c) consider clustering of variables across observational units. For our analysis, they provide several benefits compared to other modeling approaches. Given the hierarchical nature of our dataset which contains subdistricts nested within districts of Gujarat, GEEs are more appropriate than spatial autoregressive models in which spatial dependence is estimated somewhat arbitrarily based on contiguity or distance-based parameters. GEEs are also preferable to other modeling approaches that consider non-independence of data, such as mixed models with random effects, because GEEs estimate unbiased population-averaged or marginal regression coefficients, which makes the approach suitable for analyzing general patterns of inequality across subpopulations [52,56]. Mixed models with random effects, in contrast, generate cluster-specific (i.e., conditional or subject-specific) results that may not provide as reliable an inferential basis for comparing population subgroups in our study [52,62]. Additionally, GEEs are more appropriate for this study than multilevel modeling approaches since the intracluster correlation estimates are adjusted for as nuisance parameters and are not modeled [60,63].

For estimating a GEE, clusters of observations must be defined, which assumes that observations from within a cluster are correlated, while observations from different clusters are independent. Our cluster definition was based on the district within which each subdistrict is located (26 clusters of subdistricts), based on the assumption of dependence of subdistricts within a particular district in Gujarat. GEEs also require the specification of an intra-cluster dependency correlation matrix, known as the working correlation matrix [56]. The working correlation matrix structure was specified as exchangeable, since this specification assumes intra-cluster dependency to remain constant [52].

To select the best-fitting model, the GEEs were run six times for each dependent variable, based on modifying the model specifications. Since all the dependent variables were continuous, we specifically explored normal, gamma, and inverse Gaussian distributions with log and identity link functions. An identity link function assumes the dependent variable is directly predicted and not transformed, while a log link function predicts the natural logarithm of the dependent variable. We selected the normal distribution with log link function for the final GEE, since this specification yielded the lowest value of the QIC (quasi-likelihood under the independence model criterion), indicating the best statistical fit. It should be noted that although QIC fit statistics are used to select best fitting models or determine the best link function for each dependent variable, they cannot be compared across the different GEEs presented. All independent variables were standardized before inclusion in the GEE models and standardized coefficients are provided to compare the relative contribution of each variable. Two-tailed *p*-values from the Wald’s chi-squared test were used to evaluate the statistical significance of each individual variable. Finally, the multicollinearity condition index was calculated for the combination of independent variables included in each GEE. None of the models yielded a condition index higher than 5.0, indicating the absence of collinearity problems.

## 3. Results

**Figure 1:**
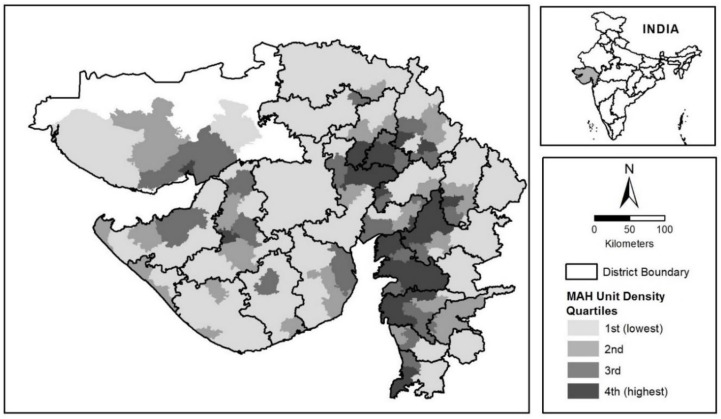
Distribution of MAH unit density by subdistrict and location of Gujarat, India.

The density of MAH units at the subdistrict level is depicted as a classified choropleth map in Figure 1, which also shows district boundaries in the state. The MAH unit density value of each subdistrict is used to group subdistricts into four quantiles. Subdistricts with the greatest MAH unit density (highest quartile or top 25%) are located primarily in three districts: Bharuch in south Gujarat, and Ahmedabad and Vadodara in central Gujarat. These three districts collectively contain 254 of 402, or about 53% of all MAH units analyzed in this study.

We began our statistical analysis by examining bivariate linear correlations between each independent variable and five MAH unit density variables, respectively. The Pearson’s correlation coefficients associated with each pair of variables are presented in Table 2. The density of all MAH units was significantly and positively correlated with population density, urban population proportion, and literacy rate, but negatively correlated with the proportion of home owning households. A similar pattern was observed for all four subcategories of MAH units in the state. The proportion of SCs or STs, however, was not significantly correlated with any of the five dependent variables.

The results from the multivariate GEEs are summarized in Table 3, Table 4 and Table 5. The first model (Table 3) used the density of all MAH units as the dependent variable. This table indicates that MAH unit density was significantly related to all independent variables (*p* < 0.05), except for literacy rate. After controlling for the effects of other explanatory variables, the density of MAH units was significantly greater in subdistricts with higher proportions of SCs, STs, and urban population, but significantly smaller in districts that were more densely populated and had a higher home ownership rate. Although literacy rate showed a positive association with MAH unit density, this relationship was not statistically significant.

For the GEEs in Table 4, MAH units were classified based on production capacity. The density of large capacity industries was significantly and positively related to the proportions of the urban and ST population, but indicated a significantly negative association with home ownership rate. For density of medium/small capacity industries, all independent variables showed a significant relationship. More specifically, the density of medium/small industries was positively related to the proportions of the urban population, SCs, STs, and literates, and negatively related to population density and home ownership rate.

The GEE models in Table 5 allowed us to compare the socio-demographic distribution of MAH units by sector. The density of private sector industries was significantly higher in subdistricts with higher proportions of the urban, SC, and ST population, and lower proportion of home ownership. In contrast, for the GEEs representing public sector industries, SC and ST proportions were not significant. Instead, the density of public industries was positively related to the urban proportion and literacy rate, but negatively associated with population density and home ownership rate.

## 4. Discussion

The statistical results of this study provide several insights on social inequalities associated with the distribution of hazardous industrial facilities in Gujarat. Overall, a greater concentration of MAH units was significantly more likely to be found in subdistricts that were more urbanized, less densely populated, contained a higher proportion of socially disadvantaged residents (both SCs and STs), and a lower proportion of home-owning households, after accounting for geographic clustering in the data. When MAH units in Gujarat were classified by capacity and sector, almost similar distribution patterns and social inequities were observed for large capacity industries (except for the SC population), medium/small capacity industries, and those belonging to the private sector, respectively. Public sector industries represent the only subcategory that did not indicate a significant statistical association with the proportions of the SC and ST population.

With regard to the socially disadvantaged groups, bivariate correlation analysis did not indicate significant associations between MAH unit density and proportion of SCs or STs. After controlling for the effects of clustering and other independent variables in our multivariate GEEs; however, we found a significantly positive relationship between the SC proportion and the overall density of MAH units, as well as the densities of medium/small capacity and private sector units. We also found density of all MAH units, as well as the densities of large capacity, medium/small capacity, and private sector units, to be significantly greater in subdistricts with a higher proportion of the ST population. These results indicate the need to more carefully understand the distribution of SC/ST groups in Gujarat to determine whether they have migrated towards the employment opportunities provided by hazardous industrial facilities or if these industries have found it easier to locate in areas where socially disadvantaged groups reside. Given that SC and ST groups in Gujarat are more likely to be found in rural areas [48,49], their significant presence in subdistricts with higher MAH unit density which are also urbanized suggests an environmentally inequitable distribution.

When variables denoting socioeconomic status are considered, literacy rate suggested a positive association with several MAH unit density subcategories, after controlling for urbanization and other explanatory variables. This could imply that MAH units tend to concentrate in areas with higher availability of educated laborers for industrial jobs. The proportion of home owners, however, indicated a consistent and significantly negative association with the overall density of MAH units and all subcategories examined. While these results suggest that economically disadvantaged residents who cannot afford to purchase a home reside near hazardous industries, this finding could also reflect lower rates of home ownership in urban subdistricts of Gujarat with fewer affordable housing options. As mentioned previously, lack of rental housing in India is viewed as an impediment to the mobility of workers who may not want to purchase a house [54]. Our results suggest that rental housing stock is coincident with highly polluted subdistricts, which points to either the high costs of home ownership around industrial facilities, or the unwillingness of those with the means to purchase housing to reside near hazardous industries. Overall, this leads to the conclusion that home ownership is a very useful variable to pursue in future analyses of distributive EJ in India.

In terms of the control variables of this study, densities of all MAH units, medium/small capacity industries, and public sector industries were found to decline with an increase in population density. This finding is similar to those reported in national-scale EJ studies conducted in India and the U.S., which demonstrate a negative association between population density and hazardous industrial pollution after controlling for urbanization [37,38,41]. With respect to Gujarat, medium/small capacity and public sector MAH units were more likely to locate in urbanized subdistricts that were sparsely populated, possibly due to these having higher availability of vacant land that were proximate and accessible to major urban centers. This result coincides with previous EJ research that has depicted sparsely populated urban areas as having a lower ability to control the presence of industrial pollution in their vicinity [41]. Large and private sector industries in Gujarat, however, are concentrated in larger urban subdistricts and do indicate a statistically significant association with population density. The extent of urbanization, as measured by the urban proportion, significantly influenced the distribution of all MAH units and the four subcategories examined, even after controlling for other socio-demographic variables. Thus, urbanization continues to attract industrialization in Gujarat despite government efforts to shift industries to rural and less populated and polluted areas [14].

Finally, it is important to consider specific limitations of our study that are related to the unavailability of more detailed information on hazardous industries and potentially affected populations. First, data on industries and industrial pollution continues to be difficult to access in the context of India despite some steps taken towards rectifying the situation through the Environment (Protection) Act 1986 and Central and State Pollution Control Boards. Thus, the quantity or quality of pollutants emitted by each MAH unit are not available and this prevents us from assessing human health risks posed by hazardous industries based on exposure and toxicity. Second, although MAH units store or transport the highest quantities of toxic chemicals and pose the greatest health risks for local residents compared to other facilities, they are not the only source of industrial pollution in Gujarat. For a more comprehensive EJ assessment, it is also necessary to analyze industries that manage smaller volumes of toxic substances, as well as facilities that are involved in the treatment, storage, and disposal of industrial hazardous waste. Third, our study is based on socio-demographic variables from the Census of India, which represent residential characteristics of subdistricts. The hazardous industries examined in this study can have adverse effects on not just where people live, but also where people work and conduct other daily activities. This implies that even if a socially disadvantaged subdistrict contains few or no hazardous industries, residents of the subdistrict could be exposed to pollution generated by these industries in non-residential locations such as places of work, education, and shopping. It is thus important to explore additional data sources, including surveys at the household level, that can provide a more fine-grained analysis of the EJ implications of industrial development in India.

## 5. Conclusions

This article contributes to distributive EJ research in India by exploring the relationship between social disadvantage and industrial MAH units in Gujarat. Our results reveal that higher urbanization and lower home ownership were strong predictors of MAH unit concentration, and that the presence of socially disadvantaged populations was significantly related to the density of all types of MAH units, except for those associated with the public sector. Since industrialization and industrial pollution are likely to continue in Gujarat, environmental policies and practices related to pollution control and waste management should incorporate EJ principles to ensure that the negative externalities of industrial development are not disproportionately distributed. More broadly, the association between SC/ST groups and potential proximity to industrial pollution needs to be further investigated in the case of India [37]. Government policies need to take account of the fact that the historical disadvantage faced by these groups may continue to be reflected in the unequal distribution of industrial pollution sources. Future research should also consider the processes that are potentially responsible for the environmentally unjust distribution of hazardous industries that would provide a basis for more effective policies to control industrial pollution and promote social justice.

## Figures and Tables

**Table 1:** Summary statistics for variables analyzed (*n* = 225 subdistricts).

Variable	Min	Max	Mean	Std. Dev.
Dependent variables:				
Density: all industries (MAH units)	0.000	0.103	0.004	0.011
Density: large capacity industries	0.000	0.085	0.003	0.008
Density: medium/small capacity industries	0.000	0.021	0.001	0.003
Density: private sector industries	0.000	0.085	0.003	0.009
Density: public sector industries	0.000	0.030	0.001	0.003
Independent variables:				
Population density (persons per square km)	28	14,360	492	1285
Proportion urban population	0.000	1.000	0.215	0.211
Proportion Scheduled Caste	0.001	0.182	0.070	0.040
Proportion Scheduled Tribe	0.001	0.981	0.181	0.298
Proportion literate	0.359	0.817	0.644	0.090
Proportion households owning home	0.450	0.991	0.892	0.083

**Table 2:** Bivariate correlations (Pearson’s *r*) between dependent and independent variables.

Variable	All MAH Units	Large Capacity	Medium or Small Capacity	Private Sector	Public Sector
Population density	0.373 **	0.331 **	0.459 **	0.346 **	0.369 **
Proportion urban population	0.438 **	0.420 **	0.439 **	0.402 **	0.434 **
Proportion Scheduled Caste	−0.014	−0.021	0.015	−0.006	−0.004
Proportion Scheduled Tribe	−0.043	−0.028	−0.073	−0.040	−0.058
Proportion literate	0.281 **	0.272 **	0.266 **	0.259 **	0.248 **
Proportion households owning home	−0.484 **	−0.515 **	−0.374	−0.414 **	−0.584 **

* *p* < 0.05; ** *p* < 0.01.

**Table 3:** Generalized estimating equations (GEEs) for predicting density of all MAH units.

Variable	Beta	Wald’s Chi-Sq.
Population density	−0.082	5.155 *
Proportion urban population	0.787	16.422 **
Proportion Scheduled Caste	0.682	3.144 *
Proportion Scheduled Tribe	1.725	19.140 *
Proportion literate	1.844	2.899
Proportion households owning home	−0.502	6.596 *
Intercept	−8.534	23.218 **
Model fit (QIC)	49.958	
N (subdistricts)	225	

* *p* < 0.05; ** *p* < 0.01.

**Table 4:** GEEs for predicting density of MAH units by capacity.

Variable	Large Capacity		Medium/Small Capacity	
	Beta	Wald Chi-Sq.	Beta	Wald Chi-Sq.
Population density	−0.071	1.973	−0.152	16.946 **
Proportion urban population	0.628	9.661 **	1.464	17.454 **
Proportion Scheduled Caste	0.511	2.651	1.585	9.291 **
Proportion Scheduled Tribe	1.454	24.360 **	3.333	12.091 *
Proportion literate	1.271	3.090	4.801	7.489 **
Proportion households owning home	−0.475	11.524 **	−0.971	6.103 *
Intercept	−7.735	59.394 **	−16.426	16.496 **
Model fit (QIC)	45.006		66.175	
N (subdistricts)	225		225	

* *p* < 0.05; ** *p* < 0.01.

**Table 5:** GEEs for predicting density of MAH units by sector.

Variable	Private Sector		Public Sector	
	Beta	Wald Chi-Sq.	Beta	Wald Chi-Sq.
Population density	−0.063	3.259	−0.185	21.304 **
Proportion urban population	0.885	18.087 **	0.970	10.619 **
Proportion Scheduled Caste	0.781	3.929 *	1.002	3.412
Proportion Scheduled Tribe	2.159	12.397 **	0.580	0.251
Proportion literate	1.838	3.755	4.092	7.689 *
Proportion households owning home	−0.460	7.306 **	−1.172	10.973 **
Intercept	−0.901	26.707 **	−15.383	17.053 **
Model fit (QIC)	39.604		55.277	
N (subdistricts)	225		225	

* *p* < 0.05; ** *p* < 0.01.
